# Effect of the second-step voltages on the structural and corrosion properties of silicon–calcium–phosphate (Si–CaP) coatings on Mg–Zn–Ca alloy

**DOI:** 10.1098/rsos.172410

**Published:** 2018-10-10

**Authors:** Jinhe Dou, Yupeng Zhao, Lu Lu, Guochao Gu, Huijun Yu, Chuanzhong Chen

**Affiliations:** 1 Shenzhen Research Institute of Shandong University, Shenzhen 518057, Guangdong, P. R. China; 2 Key Laboratory for Liquid-Solid Structural Evolution and Processing of Materials, Ministry of Education, School of Materials Science and Engineering, Shandong University, Ji'nan 250061, Shandong, P. R. China; 3 Key Laboratory of High-Efficiency and Clean Mechanical Manufacture (Shandong University), Ministry of Education, School of Mechanical Engineering, Shandong University, Ji'nan 250061, Shandong, P. R. China; 4 National Demonstration Center for Experimental Mechanical Engineering Education (Shandong University), School of Mechanical Engineering, Shandong University, Ji'nan 250061, Shandong, P. R. China

**Keywords:** Si–CaP coating, second step, micro-arc oxidation, magnesium alloy, corrosion behaviour

## Abstract

The applications of magnesium (Mg) alloys as biodegradable orthopedic implants are mainly restricted due to their rapid degradation rate in the physiological environment. In this study, Si–CaP micro-arc oxidation (MAO) coatings were prepared on a Mg–Zn–Ca alloy by a second-step MAO process at different voltages in order to decrease the degradation rate and increase the bioactivity of the alloy. The microstructure and morphology of the samples were characterized using XRD, FT-IR SEM and EDS. The degradation behaviours of samples were evaluated using electrochemical techniques, and immersion tests in simulated body fluid (SBF). The results indicate that the morphology of the Si–CaP coatings changed significantly with the increase in Ca/P ratio as the second-step voltage increased. The Si–CaP containing coating produced at 450 V could significantly decrease the degradation rate of Mg and caused a slow increase in pH of the SBF solution. The haemolysis test concluded that the coating C3 did not cause a haemolytic reaction. The corrosion resistance of Mg alloy was greatly improved with the Si–CaP coatings, and the Mg alloy with Si–CaP coating prepared at 450 V had the best corrosion resistance, which indicates that the Si–CaP coatings are promising for improving the biodegradation properties of Mg-based orthopedic implants. Haemolysis tests indicated that the Si–CaP coating prepared at 450 V conforms to the given standard (YY/T0127.1-93).

## 1. Introduction

Magnesium (Mg) and Mg alloys have been considered for use as an innovative orthopedic implant [1,2], as they possess advantages over traditional metallic materials, biodegradable polymers and ceramics [3]. Mg and Mg alloys gradually degrade *in vivo* and are eventually replaced by newly grown bone tissue after implantation, which eliminates the need for further surgery to remove the implant [4,5]. Furthermore, the mechanical properties of Mg alloys are closer to those of natural bone [6]. Moreover, Mg ions could promote bone healing due to their functional roles in bone tissues [4]. The high chemical reactivity of Mg alloys, however, leads to a loss of mechanical integrity before the tissue has healed sufficiently and new bone tissue has adequately regenerated. Therefore, the poor corrosion resistance of Mg alloys inhibits its clinical applications.

To improve the corrosion resistance of Mg alloys, alloying and surface treatments are commonly adopted. Alloying elements such as aluminium (Al) [7,8], calcium (Ca) [9,10], zinc (Zn) [11,12], etc., have been used to develop appropriate Mg alloys. It is known that Zn is an essential element in the human body and a co-factor for various enzymes in bone [13]. Ca is a major component of human bone and is essential in chemical signalling with cells [10]. However, aluminium was reported to be a risk factor for Alzheimer's disease [14]. Sun *et al*. [15] developed a new kind of ternary Mg–Zn–Ca alloy which exhibited low corrosion rate and good biocompatibility. Therefore, these Mg–Zn–Ca alloys are an excellent alternative for bone implants.

Unfortunately, the corrosion rate in the human bio-environment of these Mg alloys still could not satisfy the requirements for biomedical application. Therefore, diverse surface modification techniques [16–18] have been employed in recent years. Micro-arc oxidation (MAO) is recognized as an efficient and simple technique for biological applications of Mg alloys, because MAO coating can improve the corrosion resistance, wear resistance and biocompatibility of the Mg. Si-containing MAO coatings have been widely studied as a barrier to the corrosion of Mg alloys *in vitro* [19,20]. These coatings could enhance their biocompatibility and slow down their corrosion rate in physiological environments [20]. Ca–P coatings have also been reported to enhance cellular adhesion, proliferation and differentiation to promote bone regeneration [21]. Gu *et al*. [22] introduced Ca and P into an MAO coating on an Mg–Ca alloy and found that the corrosion resistance and surface biocompatibility of the Mg alloy were strongly improved. However, only a few studies have prepared the Si–CaP coating by the MAO method.

In this study, the Si–CaP MAO coatings were prepared by a second-step MAO process at different voltages. The degradation behaviour of Mg alloy with MAO coating in simulated body fluid (SBF) was systematically investigated. The influence of voltages on the composition and structure of MAO coatings and the role of the Si–CaP coating in preventing the corrosion of Mg in SBF are discussed in detail.

## 2. Experimental procedure

### 2.1. Material and process

A Mg–Zn–Ca alloy ingot was used and the chemical composition is as follows (in wt%): Zn 1.74, Ca 0.55 and Mg balance. Details about the preparation of Mg–1.74Zn–0.55Ca ingot alloys can be found in our previous report [23]. The ingot preparation was carried out by homogenization at 400°C for 14 h. Afterwards, the alloy was machined into a cuboid shape with dimension of 8 × 8 × 6 mm^3^ by electric spark machining. Prior to the MAO process, the specimen was pretreated as follows: it was ground successively on silicon-carbide paper up to 1000 grit, degreased by acetone, washed with deionized water and dried in warm air.

The MAO process was conducted using a 30 kW DC power supply (Unicorntech, WHD-30) in conjunction with stirring and cooling circulating systems to keep the electrolyte temperature below 30°C. A typical MAO cell, comprising a 3 l stainless steel serving as the cathode and the Mg sample as the anode, was used. A silicate electrolyte (Si-electrolyte) and a calcium–phosphate electrolyte (CaP-electrolyte) were used in this experiment. The electrolyte compositions are listed in table 1. In this study, MAO coatings on Mg alloy were prepared in a single stage as well as in two-step stages. The coatings obtained by different treatment conditions are given in table 2 and referred to as C1, C2, C3 and C4, respectively, in the following sections. In detail, during the first stage process, the sample was oxidized in Si-electrolyte at 400 V for 5 min (coating C1). In the two-step stage process, the sample obtained in the first step was taken out and cleaned and further oxidized in CaP-electrolyte at various voltages (400 V, 450 V and 500 V) for 5 min (coating C2, C3 and C4). During each stage, the applied pulse frequency, positive duty ratio and negative duty cycles were fixed as 600 Hz, 30% and 20%, respectively. After treatment, coated Mg samples were washed using running deionized water and dried in a stream of warm air.

**Table 1.** Electrolyte compositions for MAO on magnesium alloys.

electrolyte	electrolyte constituents	additive
Si-electrolyte	15.0 g l^−1^ Na_2_SiO_3_·9H_2_O	5 g l^−1^ KOH, 7 g l^−1^ NH_4_HF_2_ and 7.5 ml l^−1^ C_3_H_8_O_3_
CaP-electrolyte	7.5 g l^−1^ (C_3_H_7_O_6_P)Ca (calcium glycerophosphate), 7.5 g l^−1^ (NaPO_3_)_6_	5 g l^−1^ KOH, 7 g l^−1^ NH_4_HF_2_ and 7.5 ml l^−1^ C_3_H_8_O_3_

**Table 2.** The coatings obtained by conditions of MAO treatment for samples.

coating code	first-step solution	first-step (V)	second-step solution	second-step (V)
C1	Si-electrolyte	400	—	—
C2	Si-electrolyte	400	CaP-electrolyte	400
C3	Si-electrolyte	400	CaP-electrolyte	450
C4	Si-electrolyte	400	CaP-electrolyte	500

### 2.2. Microstructure characterizations

The surface and cross-sectional morphologies of the MAO coatings were identified using a scanning electron microscope (SEM, Hitachi S-3400N, Japan) with an energy dispersive spectroscope (EDS). The chemical composition of the coatings was characterized by X-ray diffraction (XRD, Shimadzu XRD-6100, Japan) with a Cu K*α* target. The chemical structure of the coatings was measured using infrared spectra with a Fourier-transform infrared spectrum analyzer (FT-IR, Bruker Tensor-37, Germany).

### 2.3. Immersion tests

In order to evaluate the *in vitro* bioactivity of samples, immersion tests were carried out in SBF solutions [24]. The SBF was prepared by dissolving reagent-grade 7.996 g l^−1^ NaCl, 0.350 g l^−1^ NaHCO_3_, 0.224 g l^−1^ KCl, 0.228 g l^−1^ Na_2_HPO_4_, 0.305 g l^−1^ MgCl_2_·6H_2_O, 40 ml l^−1^ HCl (1.0 M), 0.278 g l^−1^ CaCl_2_·2H_2_O, 0.071 g l^−1^ Na_2_SO_4_ and 6.051 g l^−1^ (CH_2_OH)_3_CNH_2_ in deionized water buffered at pH 7.40 with (CH_2_OH)_3_CNH_2_ and 1.0 M HCl at 36.5°C. Samples were vertically soaked in tightly sealed polypropylene bottles with an immersion ratio of 0.08 cm^2^ l^−1^. The bottles were placed in a thermostatic water bath maintained at 36.5 ± 0.5°C for 21 days. The container was placed in a digitally controlled water bath maintained at 37 ± 0.5°C for 7 days to compare the corrosion degree in each solution.

At different time points, the pH values of the solutions were measured using a pH meter. This pH meter was calibrated before each measurement. Three samples were measured for each test. Each sample was weighed before being immersed in solutions. At different immersion time of 2, 6, 12 and 18 days, the specimens were removed from the SBF, washed with chromic acid (200 g l^−1^ Cr_2_O_3_ + 10 g l^−1^ AgNO_3_) for 5 min, followed by rinsing with running distilled water and drying in warm air. Weight loss was calculated by the following equation to evaluate the corrosion rate: weight loss (%) = (*m_o_* – *m_t_*)/(At) × 100%, where m_0_ is the weight of the sample before the degradation (mg), *m*_t_ is the weight of the sample after immersion in SBF (mg), *t* is the immersion time (d) and *A* is the exposed area of sample (cm^2^). The weight of three duplicate samples was used for average values and standard deviations. In addition, both the composition and surface morphology of the coated and uncoated samples after immersion in SBF for 18 days were examined by XRD and SEM equipped with EDS.

### 2.4. Electrochemical test

To evaluate the corrosion behaviour of both coated and uncoated samples in SBF, potentiodynamic polarization tests were conducted using a Zennium electrochemical workstation (Zahner Electric Co., Germany). A conventional three-electrode cell system comprising the sample with an exposed area of 0.64 cm^2^ as a working electrode, a saturated calomel electrode as a reference electrode and a platinum plate as a counter electrode was used in this study. The polarization scan was measured from −3.0 to 1.0 V at a scan rate of 1 mV s^−1^.

### 2.5. Haemolysis test

New Zealand rabbit (male) blood (Shandong Lukang Pharmaceutical Co., Ltd., People's Republic of China) containing potassium oxalate saline solution (2 wt%) in the ratio of 20 : 1 was diluted with normal saline (4 : 5 ratio by volume). Mg and MAO coatings samples (8 × 8 × 6 mm^3^) were dipped in test tubes containing 10 ml of normal saline that were previously incubated at 37°C for 30 min. Then 0.2 ml of diluted rabbit blood was added to each test tube and the mixtures were incubated at 37°C for 60 min. Similarly, 10 ml deionized water was used as a positive control (pc) and 10 ml normal saline solution only as a negative control (nc). Next, all the tubes were removed and centrifuged for 5 min at 1200 r.p.m. and the supernatants were carefully removed and placed in a cuvette for spectroscopic analysis at 545 nm. The optical density of the supernatant fluid was calculated using a Victor1420 (PerkinElmer Company, USA). The test was repeated three times. The haemolytic properties were determined by the haemolysis ratio (Z%), which was calculated using the following equation:

$$Z\text{\%}=\frac{OD_{t}\;-OD_{\mathrm{nc}}}{OD_{\mathrm{pc}}\;-OD_{\mathrm{nc}}}\times 100\text{\%}.$$

## 3. Results

### 3.1. Chemical composition

**Figure 1.**
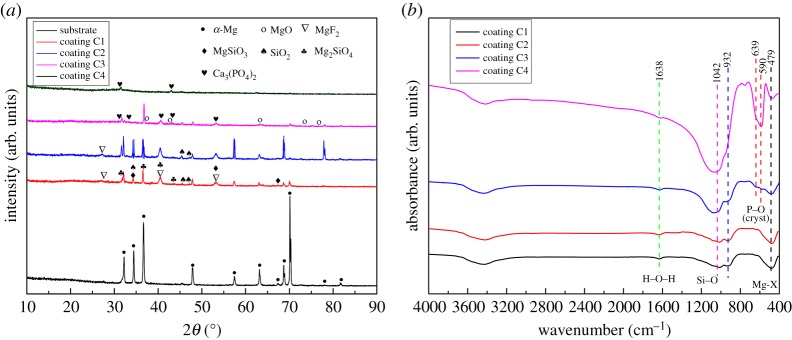
(*a*) The XRD patterns of MAO coatings and uncoated substrates and (*b*) FT-IR spectra of MAO coatings.

The XRD patterns of MAO coatings and uncoated substrates are shown in figure 1*a*. Based on XRD results, only feature peaks of α-Mg were observed in the pattern of Mg–1.74Zn–0.55Ca alloy. After the first MAO step, the coating C1 exhibited the presence of MgF_2_, MgSiO_3_, SiO_2_ and Mg_2_SiO_4_. These phase peaks were also found for the coatings C2 and C3 after the two-step MAO process. Meanwhile, new peaks of Ca_3_(PO_4_)_2_ phase could be observed in the coatings C3 and C4 but not observed in the coating C2. In addition, the relative intensity of the peaks of α-Mg in all MAO coatings reduced significantly due to the increasing coating thickness.

Figure 1*b* shows the FT-IR spectra of MAO coatings formed by the one-step and two-step MAO process on the Mg–1.74Zn–0.55Ca alloy. The FTIR spectra of coatings C1 and C2 are similar and slightly different from those of coatings C3 and C4. In all FTIR spectra of the MAO coatings, the peak at 1042 cm^−1^ is ascribed to Si–O (s, asym) asymmetric stretching mode. The SiO*_x_^n^*^−^ (SiO_4_^2−^and SiO_3_^2−^) brand is present at 932 cm^−1^ [25]. The band observed at 479 cm^−1^ is due to Mg-X (X=O or F) stretching vibrations. The band at 1638 cm^−1^ is related to the H–O–H bending mode of crystal water. For coatings C3 and C4, phosphate absorption bands at 639 cm^−1^ and 590 cm^−1^ appear. The double peaks at about 600 are a characteristic feature of phosphate in crystalline phases [26].

### 3.2. Coating surface and interface characterizations

**Figure 2.**
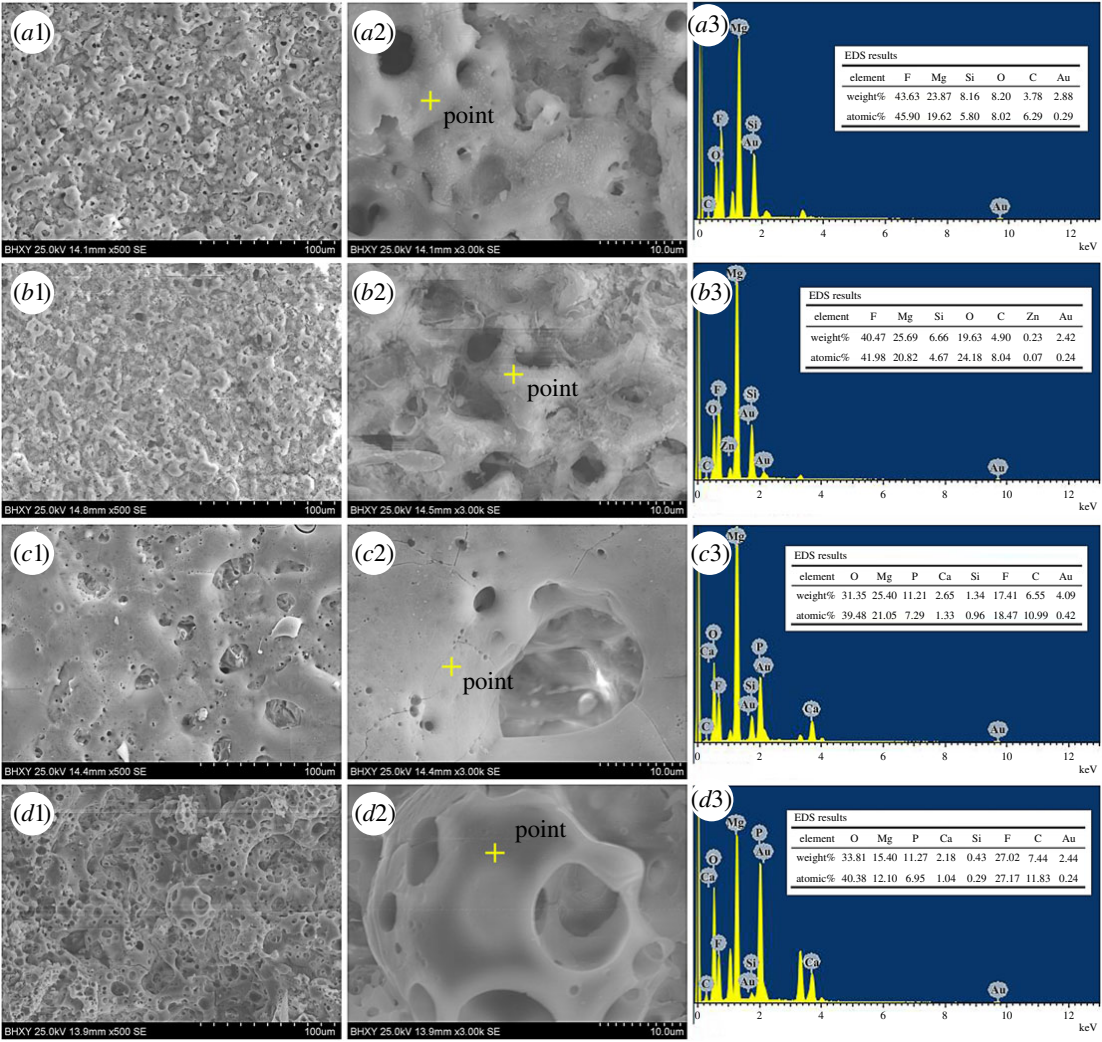
Surface morphology and EDS analysis of MAO coatings formed by (*a*) first-step and (*b*–*d*) by second-step MAO process with different voltages: (*b*) 400 V, (*c*) 450 V, (*d*) 500 V.

Figure 2 presents the surface morphology and EDS analysis of the coatings formed by one- and two-step MAO processes on Mg–1.74Zn–0.55Ca alloy. The left column presents the morphology seen at low magnification (500×) while the middle column presents the morphology at high magnification (3000×). All the coatings exhibited a typically porous and volcano-like structure, while no visible cracks could be detected in the SEM micrographs, indicating that these MAO coatings have outstanding uniformity. In addition, a large number of pancake-like disorderly micro-pores were observed on the rough MAO coating. The micro-pores are considered as the ‘footprints’ of the plasma discharge channels, through which the molten oxide and gas bubbles are probably ejected and reach the coating/electrolyte interface during the plasma-generating process [27]. The molten oxide finally sinters and deposits on the coating surface, thus contributing to the formation of oxide coatings [28].

From figure 2*a*1 and *a*2, micro-pores of different diameters (0.5–5 µm) demonstrate that different discharge types, including strong, moderate and small, contribute to the formation of the oxide coating. The morphology of coating C2 (figure 2*b*1) is similar to that of coating C1 (figure 2*a*1) at 500× magnification. However, when increasing the magnification from 500 to 3000×, the C2 coating surface (figure 2*b*2) turns out to be uneven, fairly rough and even to consist of whisker-like grains. When the second voltage increases to 450 V, the C3 coating surface remains a similar porous structure and becomes smooth with no grains compared with the C2 coating. Moreover, several cracks are observed on the coated surface (figure 2*c*1 and *c*2), which could be correlated to the thermal stress generated under high pressure and temperature during the MAO process. As the second voltage continues to increase, it can be seen from figure 2*d*1 and *d*2 that the morphology of the coatings changes greatly with large pores and cracks on the surface.

The EDS analysis of MAO coatings is presented in the right column of figure 2. In the C1 and C2 coating, F, Mg, Si, O and C elements were detected as components of the MAO coatings, with F and Mg as the major constituents (figure 2*a*3 and *b*3). The EDS analysis of the C3 (figure 2*c*3) and C4 (figure 2*d*3) coating revealed that the coating surface was mainly composed of O, Mg and F, but incorporated with small amounts of Ca, P, Si and C. The presence of Ca and P in the EDS analysis indicated that these elements were incorporated into the coating in the second MAO stage.

**Figure 3.**
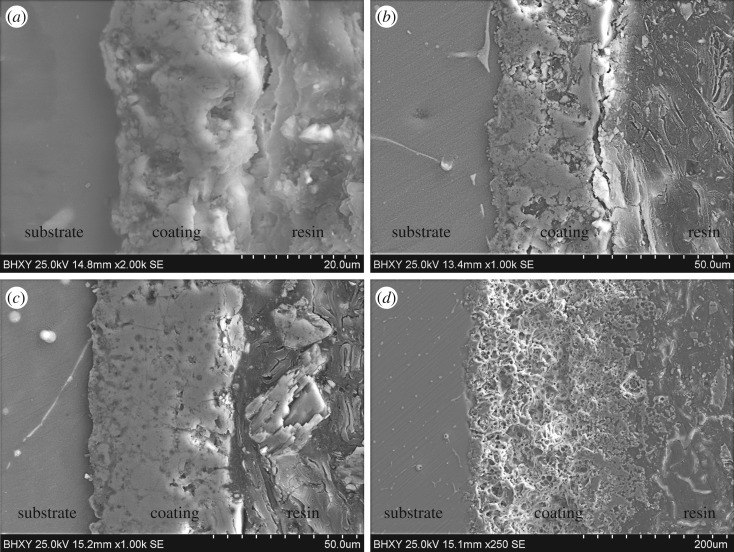
Cross-sectional SEM images of MAO coatings formed by (*a*) first-step and (*b*–*d*) by second-step MAO process with different voltages: (*b*) 400 V, (*c*) 450 V, (*d*) 500 V.

Cross-sectional SEM images of MAO coatings using different treatment conditions are shown in figure 3. As can be seen, the MAO coatings have a significant amount of connected micro-pores, cracks and other structural defects. However, inhomogeneous defects did not penetrate into the whole coating. In addition, no obvious voids or cracks were observed between the MAO coatings and the magnesium substrate, suggesting good adhesion between the coating and the Mg substrate.

The coating C1 formed by the first-step MAO process (figure 3*a*) was about 20 µm thick. In addition, the coating contained big voids and cracks. This is consistent with the coating C1 surface morphology, figure 2*a*, which shows a high density of open (un-sealed) channels at the centre of the ‘pancake’ structures, which could extend to the coating–substrate interface. The coating C2 formed by the second-step MAO process (figure 3*b*) was much thicker and more compact compared with the coating C1 (figure 3*a*), with a thickness of about 40 µm. As the voltage of the second-step process increased, the coating thickness increased. The thickness of the coatings was in the range of 70–90 µm and 210–250 µm for the samples treated for 450 and 500 V, respectively. The coating C3 was compact and had little voids. However, when the voltage was increased to 500 V, the resulting coating C4 (figure 3*d*) was almost completely composed of a porous layer.

### 3.3. *In vitro* corrosion and degradation

#### 3.3.1. Electrochemical measurements

**Figure 4.**
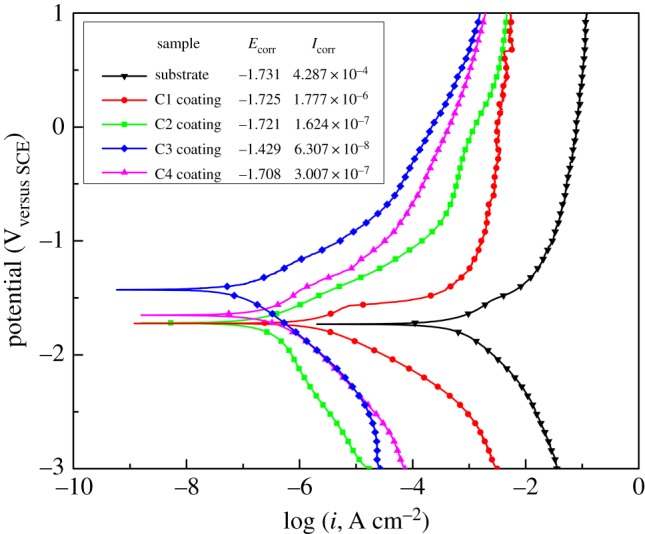
Potentiodynamic polarization curves of the samples in SBF solution.

Figure 4 displays the potentiodynamic polarization plots (Tafel plots) of the untreated and as-coated Mg alloy in SBF solution at 37°C. The corrosion potential (*E*_corr_, V) and current density (*i*_corr_, µA cm^−2^) values are derived from potentiodynamic polarization curves according to Tafel extrapolation and also given in figure 4.

In general, materials that show a higher *E*_corr_ and lower *I*_corr_ tend to offer better corrosion resistance, and vice versa [29,30]. As exhibited in figure 4, all the MAO coatings largely improved the *E*_corr_ values from −1.731 V (bare Mg alloy) to −1.721 V to −1.429 V (coating C3), indicating the ability of the MAO coating to counter the rapid corrosion of bare Mg–Zn–Ca alloy. Furthermore, the observed *I*_corr_ values were reduced by two orders of magnitude for the coating C1 formed by the first-step MAO process compared to the bare Mg–Zn–Ca alloy. In addition, results showed that the order of the *I*_corr_ of the coatings was C1 > C4 > C2 > C3. The above results demonstrated that the coating C3 was more effective in blocking the infiltration of aggressive ions to the bare Mg–Zn–Ca alloy, therefore resulting in better corrosion protection.

#### 3.3.2. *In vitro* immersion tests

**Figure 5.**
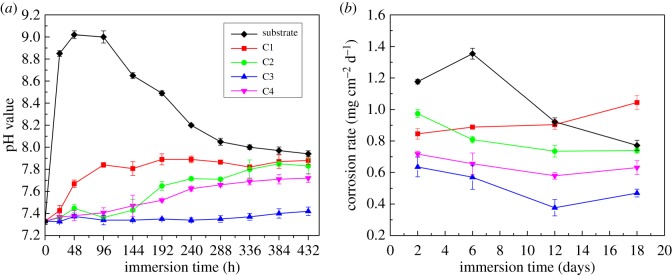
Variance of pH value (*a*) and corrosion rate (*b*) of samples tested in SBF solution.

The immersion test in SBF solution was performed for 18 days at 36.5°C in order to examine corrosion behaviour of the MAO coatings and bare Mg–Zn–Ca alloy samples. The changes in the pH value of SBF solution at different immersion times are presented in figure 5*a*. As shown in the results, the control groups (bare Mg–Zn–Ca alloy) exhibited a rapid rise in pH from 7.33 to over 9.00 within the first 2 days of immersion, and then sharply decreased until reaching 7.94 ± 0.03 at the end of the testing period. However, all the MAO coatings showed significantly lower pH values than the bare Mg–Zn–Ca alloy during the immersion time. The pH of the SBF solution containing coating C1 reached 7.85 in 4 days and stabilized afterwards. With regard to the coatings C2 and C3, the pH increase was slower. For coating C4, the pH continued to increase in the first 12 days and then stabilized. Moreover, for the coating C3 after the initial small increase in pH during the first 2 days, a much lower pH value (about 7.35) was observed, which eventually reached 7.42 ± 0.04 at the end of day 18. The above result of pH values demonstrated that the MAO coatings showed better capability of preventing the medium from becoming unacceptably alkaline to local tissues than the bare Mg alloy.

The corrosion rates of MAO coatings and bare Mg–Zn–Ca alloy samples are shown in figure 5*b*. As can be seen, the corrosion rate of the MAO coatings formed by the second-step process is lower than that of Mg alloy at every immersion time while the corrosion rate of the coating C1 is lower than that of Mg alloy until immersion for 12 days. Moreover, the coating C3 has the lowest corrosion rate, which indicates that the coating C3 is synthetically the most protective. Besides, the immersion time has a large influence on the corrosion rate. As can be seen in figure 5*b*, the corrosion rates of the MAO coatings formed by the second-step process decrease during the initial 12 days and then increase slightly during the rest of the procedure. For the bare Mg alloy, the corrosion rate increases sharply from 1.18 to 1.35 mg cm^−2^ d^−1^ during the initial 6 days; after that, the corrosion rate decreases sharply to 0.77 mg cm^−2^ d^−1^. However, in the case of the coating C1, the corrosion rate always increases with an increase in the immersion time.

#### 3.3.3. *In vitro* bioactivity

**Figure 6.**
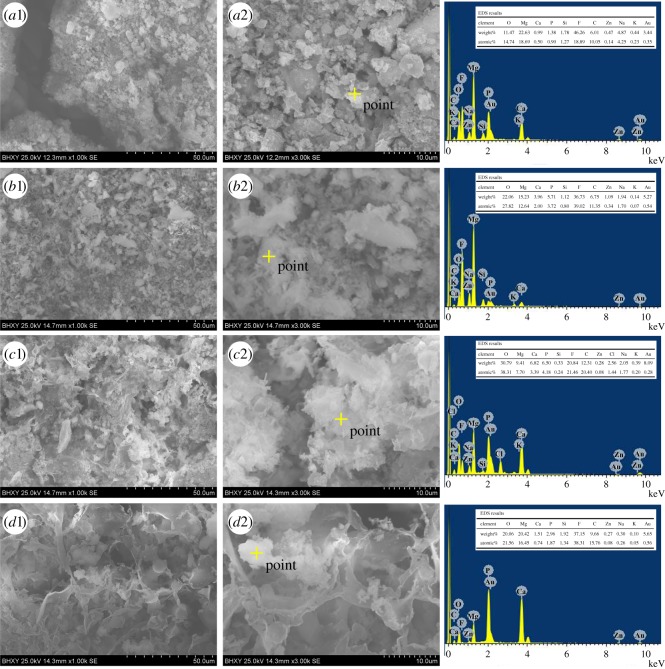
SEM morphology and respective EDS element analysis of coating samples after 18 days immersion in the SBF solution: (*a*1)(*a*2) coating C1, (*b*1)(*b*2) coating C2, (*c*1)(*c*2) coating C3 and (*d*1)(*d*2) coating C4.

Figure 6 shows the SEM micrographs of MAO coatings after 18 days of immersion in the SBF solution at 36.5°C. As shown in figure 6, the original surface morphology of all MAO coatings has disappeared and white layers are found deposited on the surface as degradation products. Meanwhile, except for the coating C1, the cracks resulting from corrosion could hardly be observed in the surface morphology of the other samples after the immersion test. From figure 6*a*1 and *a*2, it can be observed that the coating C1 surface morphology has been corroded and a few cracks have appeared. In addition, precipitates with a spherical-like structure can be observed on the coating surface. According to figure 6*b*1 and *b*2, the total surface of coating C2 has been covered by white particles with a spherical-like structure. As shown in figure 6*c*1 and *c*2, the density of deposited white particles on the surface of coating C3 is more than that of the other samples. However, a continuous and porous morphology of the white framework particles were observed on the coating C4 surface (figure 6*d*1 and *d*2). EDS analysis was also applied to investigate the elemental composition of the MAO coatings after 18 days of SBF immersion, as shown in figure 6. The EDS patterns of all selected points in figure 6 revealed that the corrosion products were mainly composed of O, Mg, Ca, P, Si, F and C. Weak peaks of Na, K and Cl were also observed, which presumably were induced by the precipitation of NaCl and KCl salts from the SBF.

**Figure 7.**
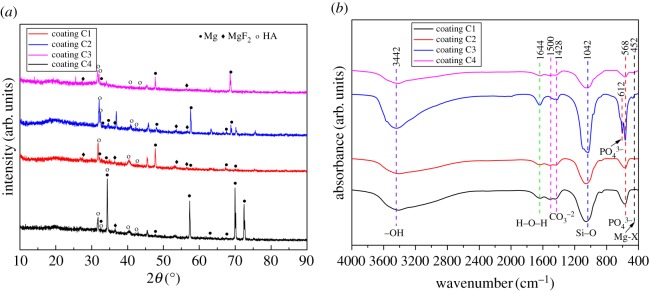
XRD and FTIR spectra of the MAO coatings after 18 days of immersion in the SBF solution.

To verify the phase and chemical composition of the coatings after 18 days of immersion in the SBF solution, X-ray diffraction and FTIR spectra were used, and the results are shown in figure 7. As shown in the XRD spectra (figure 7*a*), the typical diffraction peaks of pure Mg appeared in the XRD patterns of the MAO coatings immersed in SBF solution for 18 days. It is noteworthy that for the coating C1 the intensity peaks of Mg were stronger than for the other coatings. However, the peaks corresponding to Mg(OH)_2_ were not clearly observable for all MAO coatings, which was routinely reported under *in vitro* environments [29,31]. Although Mg(OH)_2_ is slightly soluble in SBF, rigorous degradation occurred in aqueous physiological media, as in aqueous solutions with high chloride concentrations Mg(OH)_2_ reacts with Cl^−^ to produce highly soluble MgCl_2_ [32,33]. In addition, new characteristic peaks for hydroxyapatite (HA, Ca_10_(PO_4_)_6_(OH)_2_) that mimic the bone phases were detected on all MAO coatings after the immersion test. The results indicate that MAO coatings have great apatite-forming ability after soaking in SBF solution.

FT-IR was used to detect the chemical groups of the MAO coatings (shown in figure 3). Accordingly, all the MAO coatings exhibit typical peaks of the phosphate region (900–1200 cm^−1^) of HA. The shoulder peak at 1042 cm^−1^ is associated with part of amorphous HA. Furthermore, the doublet bands presented at 612 cm^−1^ and 568 cm^−1^ correspond to the bending vibration of P–O, which is indicative of HA [34,35]. The peak at 1644 cm^−1^ (bending mode) is associated with H_2_O and the adsorption bands at 1500 and 1428 cm^−1^ are attributed to carbonate ions [36]. The FT-IR spectra results confirm that the apatite formed on MAO coatings has a carbonated structure.

### 3.4. Haemocompatibility

**Figure 8.**
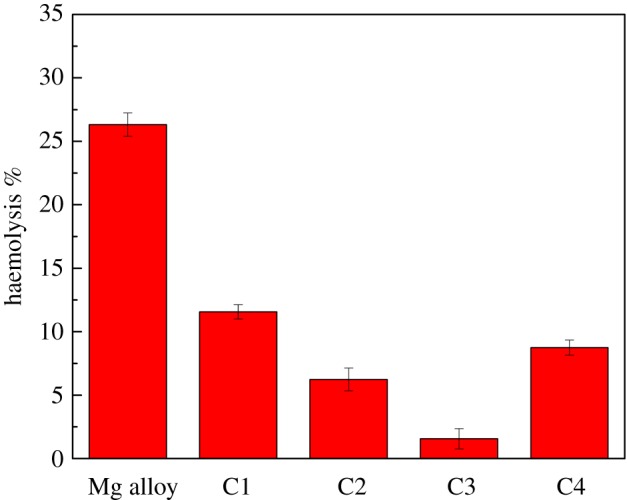
Haemolysis ratio of Mg alloy and MAO coating samples.

Figure 8 shows the haemolysis test results for the Mg alloy and MAO coating samples. It was found that the haemolysis ratio of Mg–Zn–Ca alloy is higher than that of the MAO coating samples. For the coatings, the haemolysis ratio of the coating C3 is 1.56% (*Z* < 5%), which indicates that the coating C3 conforms to the given standard (YY/T0127.1-93).

## 4. Discussion

### 4.1. Effect of voltage on the coating formation

The morphology and other properties of MAO coatings are found to be affected by many factors such as current density, voltage, pulse frequency, treatment time, duty cycle, electrolyte solution and post treatments [37,38]. In the present work, a porous and uniform Si–CaP coating was successfully prepared on the Mg alloy by a two-step MAO process, which was confirmed by the results of FT-IR, XRD, SEM and EDS analyses (figures 1 and 2). The results have shown that the effect of voltage on the formation of a Si–CaP coating on the Mg alloy is quite dramatic. Moreover, the coating C3 deposited at 450 V exhibited the best surface quality with a relatively smooth and homogenous microstructure. In previous studies [19,20], it was found that the increase in voltage could cause an increase in the pore size and decrease in the number of pores in MAO coatings. According to the change of surface morphology with voltage (figure 2), the second step was a process of new MAO coating formation. This process is similar to the one-step MAO process, in which both the dissolution of magnesium and development of new coatings are involved. Thus, the effect of voltage on the formation of Si–CaP coating is speculated as follows.

After the first-step MAO process, the coating C1 was immersed in CaP-electrolyte for the second MAO process, resulting in a competition between the old coating dissolution and the new coating formation [39]. Part of the old coating formed in the Si-electrolyte may be dissolved in the CaP-electrolyte. For example, the following reaction may take place:

$$\mathrm{MgO}+H_{2}O\to Mg^{2+}+OH^{-}$$

When the applied voltage was lower than the dielectric breakdown voltage, the new coating formation was restricted and the dissolution speed may be higher than the development speed. The breakdown voltage of the second-step MAO process in the present study should be lower than 400 V because the coating C2 was thicker than the coating C1. When the voltage exceeds the breakdown potential, sparks can be seen on the sample surface which always appear at the thinner or weaker spots and move to other thin or weak spots in the coating [40,41]. At the sparking areas, the local temperature is estimated to be over 1000°C and the pressure can reach 10^2^–10^3^ MPa [42]. Under such high temperature and pressure, the coating formed in the first step melted and erupted into the electrolyte. Therefore, in the initial stage of the second-step MAO process, the old coating formation was destroyed. This process was dominated by the dissolution and destruction of the old coating.

When the voltage continued to rise, the breakdown of the old coating became easy. The melting and the dissolution of Mg substrate increased the Mg^2+^ ion concentration at the coating/electrolyte interface. When enough Mg^2+^ ions accumulated at the interfaces, the Mg^2+^ ions would combine with anions from the CaP-electrolyte such as F^−^, OH^−^, PO_4_^3−^ or Ca^2+^ to form compounds on the sample surface. Under such high temperature and pressure, the compounds melted and erupted into the solution along discharge channels, and a porous structure was formed when the melt was cooled down by the electrolyte. The coating continuously grows in this way until more energy required for further breakdown of the coating cannot be provided. As a result, the coating thickness gradually increased with the oxidation time (figure 3), and the surface morphology was mainly attributed to discharge behaviours in the later oxidation stage. At 500 V, large severe sparks with high density and intensity were observed, along with violent gas evolution and rapid rise in temperature near the anode [43]. Besides, the gas bubbles thrown out of micro-arc discharge channels were responsible for micropore generation [44], while the formation of microcracks resulted from the intense thermal stress generated during cooling [45]. In contrast, small, fine and uniformly distributed sparks should lead to a compact structure (figure 2*c*), as was obtained at 450 V.

### 4.2. Alkali effect

The rapid corrosion rates of magnesium in the physiological environment greatly limit its use in clinical applications [38]. Furthermore, the rapid degradation rate of Mg alloys results from a remarkable increase in local pH value of body fluid which may even lead to an alkaline poisoning effect in the human body [32]. Previous studies [18,20,46] have been conducted to reduce the rapid degradation rate. This study evaluates the variance of pH value of the samples in SBF solution (figure 5*a*). The pH values of the solution with the coating C1 changed greatly, which would have a severe effect on the local physiological condition. The reasons for the increase in pH value may be the dissolution of MgO, increasing the amount of OH^−^ in the SBF solution [20,47]. The following reaction may take place:

4.1 $$\mathrm{MgO}+H_{2}O\to \mathrm{Mg}{\left(\mathrm{OH}\right)}_{2}$$

and

4.2 $$\mathrm{Mg}{\left(\mathrm{OH}\right)}_{2}\to Mg^{2+}+2OH^{-}.$$

By contrast, the pH values of the solution corresponding to the coating C3 increased much slowly. This result is probably caused by the degradation of Mg_2_SiO_4_ (equation (4.3)) and Ca_3_(PO_4_)_2_ [48] (equation (4.4)), which neutralizes the hydroxyls released during the corrosion of MgO (equations (4.1) and (4.2)). Therefore, the coating C3 protected the substrate from quick dissolution into the SBF solution, which indicated the coating C3 was physiological environment-friendly.

4.3 $$Mg_{2}\mathrm{Si}O_{4}+2OH^{-}\to \mathrm{MgSi}O_{4}^{\;\;\;2-}+\mathrm{Mg}{\left(\mathrm{OH}\right)}_{2}\;$$

and

4.4 $$Ca_{3}{\left(PO_{4}\right)}_{2}+H_{2}O\to (Ca_{5}{\left(PO_{4}\right)}_{3}(\mathrm{OH}))+2Ca^{2+}+2\mathrm{HP}O_{4}^{\;\;\;2-}.$$

### 4.3. Degradation performances

The degradation behaviour of the coated Mg alloy in the physiological environment is greatly dependent on the initial structure, composition of the coating and apatite-forming ability [49,50], and subsequently affects the biocompatibility and bioactivity of the coated Mg alloy [51].

In this study, the Si-containing coating became more compact (figure 3*c*) by a second-step MAO process. Additionally, Ca_3_(PO_4_)_2_ is chemically stable in the physiological environment. MgO is a common phase in the MAO coating, which is not stable in the neutral electrolyte. Thus, the second-step MAO process will alter the structure and phase composition of the Si-containing coating.

Previous *in vitro* studies [20,43] have shown that in the initial immersion period the pH elevation around the coating due to the degradations of the MgO in the coating could contribute to the apatite formation

4.5 $$5Ca^{2+}+3PO_{4}^{\;\;\;3-}+OH^{-}\to (Ca_{5}{\left(PO_{4}\right)}_{3}(\mathrm{OH})).$$

Although the pH value of the Si–CaP coating produced in this study changed a little, a uniform apatite deposition layer has also been observed on the surface as presented in figure 6*c*1 and *c*2. This is related to the presence of Ca_3_(PO_4_)_2_ and Mg_2_SiO_4_. As the Si–CaP coating was soaked in SBF, the MgO and Ca_3_(PO_4_)_2_ phases would react continually with H_2_O (see equations (4.1) and (4.4)). Thus, the Ca^2+^ and HPO_4_^2−^ were concentrated in the interface between the Si–CaP coating and SBF solution. Higher Ca^2+^ concentration could induce the apatite formation on the sample. Furthermore, some other ions in the SBF solution, like HPO_4_^2−^, CO_3_^2−^ etc., would promote the formation of apatite nuclei. In addition, a large number of the silanol (Si-OH) groups formed on the Si–CaP coating surface from the reactions of the hydration products of Mg_2_SiO_4_ with H_2_O. The formation of Si-OH groups has an important effect on the nucleation and amount of HA from the SBF solution that would promote the formation of apatite nuclei [26,52,53]. Moreover, the Si–CaP coating with micro-pores and large surface area could significantly accelerate the ion exchange between the coating surface and the SBF solution, which would be beneficial for the fast nucleation of apatite. Once the nuclei were formed, the apatite crystals would continuously grow, because the SBF would be highly supersaturated with respect to apatite, and finally the HA deposition layer would be formed.

## 5. Conclusion

In order to improve the corrosion resistance and bioactivity of Mg–Zn–Ca alloy, in the present work an Si and an Si–CaP containing coating were prepared on a Mg–Zn–Ca alloy via the one-step and the second-step MAO processes. The following conclusions can be drawn:

(1) The Si–CaP containing coating was produced by a second-step MAO process which was a competition between the old coating dissolution and the new coating. The morphology of the Si–CaP coatings changed greatly with large pores and cracks with increase of the second-step voltage.
(2) The *in vitro* bioactivity assessments showed that the bioactivity of the Si–CaP containing coatings was significantly better than that of Mg–Zn–Ca alloy. After the specimens were immersed in SBF solution for 18 days, the bone-like apatite layer consisting of spherical-like clusters was deposited on the Si–CaP containing coating surface.
(3) The Si–CaP containing coating produced at 450 V could significantly decrease the degradation rate of Mg and caused a slow increase of the pH of the SBF solution.

## Supplementary Material

Figure S1. The hemolysis test of samples: (a) the substrate; (b) the coating C1, C2, C3

## Data accessibility

The datasets supporting this article have been uploaded as part of the electronic supplementary material.
