# Supplementary material for: Effect of the second-step voltages on the structural and corrosion properties of silicon–calcium–phosphate (Si–CaP) coatings on Mg–Zn–Ca alloy
Source: R Soc Open Sci. 2018 Oct 10;5(10):172410. doi: 10.1098/rsos.172410 (PMC6227930; doi:10.1098/rsos.172410)
Supplement: Figure S1. The hemolysis test of samples: (a) the substrate; (b) the coating C1, C2, C3 [file rsos172410supp1.docx]

**Supporting information**

Jinhe Dou, Yupeng Zhao, Guochao Gu*, Chuanzhong Chen*

Key Laboratory for Liquid-Solid Structural Evolution & Processing of Materials, Ministry of Education, School of Materials Science and Engineering, Shandong University, Ji’nan 250061, Shandong, PR China

**Address reprint requests to:** Chuanzhong Chen, Ph.D.

**Address:** Jinan 250061, Jingshi Road # 17923, Shandong, China

**Fax:** +86-531-88395991

**Tel:** +86-531-88395991

**E-mail address:** [czchen@sdu.edu.cn](mailto:czchen@sdu.edu.cn) (C.Z. Chen)


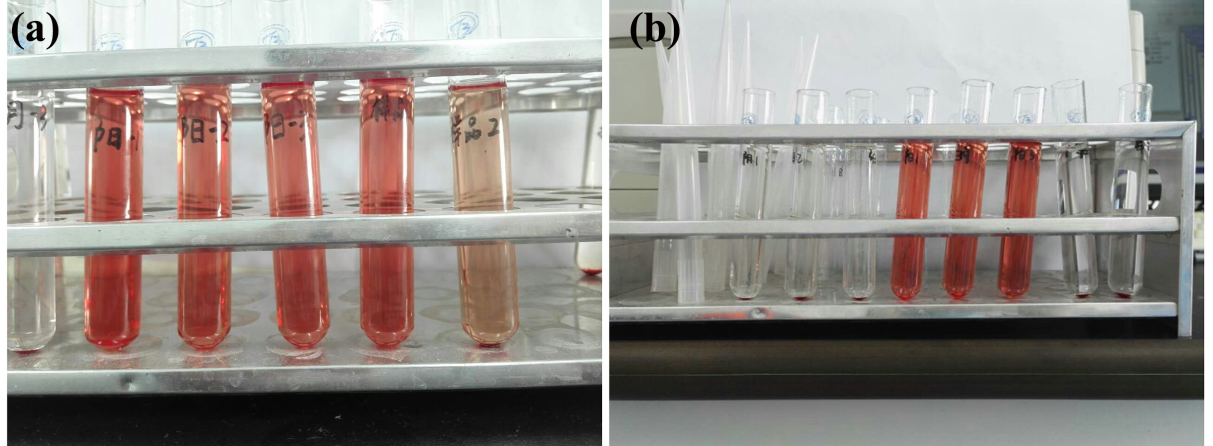


**Figure S1.** The hemolysis test of samples: (a)the substrate; (b) the coating C1, C2, C3
